# Space exploration as a catalyst for medical innovations

**DOI:** 10.3389/fmed.2023.1226531

**Published:** 2023-07-19

**Authors:** Julia Scarpa, Scott Parazynski, Gary Strangman

**Affiliations:** ^1^Department of Anesthesiology, New York Presbyterian Hospital, Weill Cornell Medical Center, New York, NY, United States; ^2^Fluidity Technologies, Inc., Houston, TX, United States; ^3^Department of Psychiatry, Harvard Medical School, Boston, MA, United States; ^4^Translational Research Institute for Space Health, Baylor College of Medicine, Houston, TX, United States

**Keywords:** aerospace, healthcare delivery, innovation, medical technology, performance optimization, remote healthcare

## Abstract

Aerospace research has a long history of developing technologies with industry-changing applications and recent history is no exception. The expansion of commercial spaceflight and the upcoming exploration-class missions to the Moon and Mars are expected to accelerate this process even more. The resulting portable, wearable, contactless, and regenerable medical technologies are not only the future of healthcare in deep space but also the future of healthcare here on Earth. These multi-dimensional and integrative technologies are non-invasive, easily-deployable, low-footprint devices that have the ability to facilitate rapid detection, diagnosis, monitoring, and treatment of a variety of conditions, and to provide decision-making and performance support. Therefore, they are primed for applications in low-resource and remote environments, facilitating the extension of quality care delivery to all patients in all communities and empowering non-specialists to intervene early and safely in order to optimize patient-centered outcomes. Additionally, these technologies have the potential to advance care delivery in tertiary care centers by improving transitions of care, providing holistic patient data, and supporting clinician wellness and performance. The requirements of space exploration have created a number of paradigm-altering medical technologies that are primed to revitalize and elevate our standard of care here on Earth.

## 1. Introduction

Technological development that supports space exploration often translates to novel and cutting-edge tools for Earth-based use. The National Aeronautics and Space Administration (NASA) is interested in mitigating identified Risks (1) associated with living in space, and, while some are space-specific, many have readily-identifiable terrestrial counterparts. The requirements of space exploration have created a number of paradigm-altering medical technologies that can elevate our standard of care here on Earth across all populations and care delivery models.

In this Perspective, we delineate the complexities of medical management during spaceflight that motivate such extensive innovation in medical technologies. Then we discuss the overlapping needs between space exploration and terrestrial environments that allow for prime translation of these technologies across the two seemingly disparate settings, including historical examples of successful technological cross-pollination. Subsequently, we explore broad categories of innovative aerospace-derived medical technologies, with representative examples. Finally, we propose high-priority terrestrial clinical applications for these technologies across in- and out-of-hospital settings, including in under-resourced areas; we also address critical touchpoints for enhancing clinician wellness and performance with these technologies. Overall, we provide a roadmap to spur clinician-leaders in spearheading collaborations with aerospace medical technology groups so that they may leverage their respective expertise to enhance patient, clinician, and astronaut health.

## 2. Physiologic consequences of spaceflight

Long-duration spaceflight results in significant changes to 1 g-dependent human physiology. While these changes are covered in detail elsewhere (2, 3), they encompass all organ systems and have variable trajectories, plateaus, degrees of reversibility, and long-term health effects. Their complex, incompletely-characterized nature has catalyzed extensive medical technology development for both research and medical care provision during spaceflight. For context, we present a very brief, generalized overview here (Supplementary Figure S1).

Spaceflight-associated neuro-ocular syndrome (SANS) is an important example: it is a syndrome never seen on Earth that affects astronauts’ vision. It is thought to be related to cephalad fluid shifts occurring in the absence of gravity, which produces alterations in brain and eye structure and function (4). Airway edema, headache, and sinus fullness occur secondary to cephalad fluid shifts too. Cognition and mental health, while not overtly affected by microgravity *per se*, are highly dependent on stress, sleep, and social–emotional factors, all of which can be disrupted by spaceflight. Additionally, radiation exposure is known to accelerate cataract formation and increases malignancy risk (5). Space-related motion sickness arises due to mismatched sensory inputs disrupting normal vestibular function (6).

From the cardiovascular perspective, cardiac output and stroke volume initially increase upon exposure to microgravity, but subsequently decline, along with cardiac muscle mass (7, 8). Peripheral vasodilation predominates and is exacerbated by changes in autonomic receptor sensitivities, endothelial changes, and vascular remodeling (9, 10). Grossly, pulmonary mechanics improve (11). Cellular and humoral immunity becomes compromised (12). This is compounded by radiation-induced genetic changes that increase microbial virulence (13). Finally, even despite vigorous exercise, bone density and muscle mass decline. Increased urinary calcium from bone resorption coupled with decreased diuresis increases the incidence of renal stones (14). Ultimately, there is still much to be learned about human physiology in this extreme environment.

## 3. Medical technology for space is well-suited to terrestrial adaptations

The challenging nature of spaceflight makes it an ideal catalyst for innovation in care delivery for remote terrestrial environments (Table 1). These sites frequently coincide with care delivery for patients requiring active monitoring and rapid diagnosis and therapy for potentially life-threatening conditions, like traumatic injuries and toxin exposures. Furthermore, clinicians operating in these conditions are often fatigued and under duress, as might be expected in deep space. However, even in well-resourced environments, monitoring, diagnosis, and therapeutics for serious conditions could be improved by facile, assistive technologies, especially in emergency, perioperative, and critical care areas.

**Table 1:** Health risks of space exploration, their terrestrial counterparts, and technological countermeasures.

**Medical concern**	**Health risks in deep space**	**Comparable scenarios on earth**	**Technological countermeasures**
Remote monitoring, early detection, and treatment of mental health and cognitive issues	Stress-, fatigue-, and radiation-induced changes in mental health and cognitive functions, including attention, processing speed, and performance	At-risk patients with limited healthcare access Follow-up care for psychiatric and neurologic diseases Mental health wellness for clinicians, especially in high-stress environments Non-pharmacologic options to limit side effects of traditional treatments	Wearable monitors (15, 16) Contactless monitors (17–20) TRISHA (21), AMRA (22) Telemedicine, telementoring of Crew Medical Officer (CMO), consultancy medicine Wearable therapeutics (23–25)
Remote monitoring, early detection, and treatment of non-emergent medical conditions	Vital signs monitoring Loading injuries Pain syndromes Renal stones Vascular flow alterations	At-risk patients with limited healthcare access Improved prehospital care Timely in-hospital care escalation/de-escalation Early, safer mobility to speed recovery Closer, safer discharge follow-up Hospital-at-home model Medical provider wellness and injury prevention Disaster care and triage	Portable imaging (26–30) and blood analysis (31–37) Wearable detection of physiologic signals (15, 16, 38, 39) and biologic compounds (40, 41) Contactless acquisition of vitals and behavior (17–20, 42–44) Contactless load detection (45) Ultrasound-based therapy (46–48) Wearable therapeutics (23–25, 49–53) ISRU with synthesis of pharmaceuticals (54–56) JITT (57, 58), telemedicine, telementoring, and consultancy medicine
Treatment of medical emergencies	Bone fractures Cardiac arrest Hemorrhage Intra-abdominal pathology Respiratory compromise Sepsis Trauma	Improved prehospital diagnosis and treatment Natural and humanitarian disasters Rural, remote, and developing areas Improved transfers of care	The above technologies for non-emergent care, plus: Crystalloid regeneration (59) Shelf-stable blood products (60) Integrated care documentation and delivery modules (61) Mixed-reality software (62, 63) and AI-CDS (21, 22, 30)
Pharmaceutical availability	Radiation-induced degradation and toxicity Limited shelf-life Limited re-supply	Drug shortages Orphan drugs Disaster supplies and re-supply	ISRU with synthetic biochemistry (54–56)
Health decrements and performance errors from fatigue, stress, and sleep loss	Circadian disturbances, high stress, close quarters, and decreased sleep while in space Small and highly interdependent crew	High-stress environments requiring heightened vigilance (e.g., ED, OR, PACU, ICU, disaster care) Medical trainees Providers on 24+ hour shifts or overnight coverage	Wearable polysomnography (15, 16) Wearable detection of stress biomarkers (40) Contactless stress detection to allow for early intervention (17–20) Non-pharmacological sleep enhancement (24, 25, 50–53)
Medical care delivery by non-experts	CMO cannot be trained in all relevant medical subspecialties Care of the CMO by other crew-members	Pandemics Natural and humanitarian disasters Rural, remote, and developing areas	AI-CDS (21, 22, 64) Autonomous and semi-autonomous systems (21, 22) JITT (57, 58), telementoring, consultancy medicine
Team cohesion and performance	Psychosocial stressors resulting from long-duration spaceflight Small and highly interdependent crew	High-stress environments requiring heightened vigilance, including ED, OR, PACU, and ICU Improved safety for patient hand-offs	Wearable detection of stress biomarkers (40) Contactless sensors (17–20) TRISHA (21), AMRA (22)
Radiation exposure	Cosmic radiation accelerates disease and increases malignancy risk	Nuclear disasters Workplace-related exposures Mitigating side effects of radiation therapy	Radioprotective oral and parenteral pharmaceuticals (65–71)

The health risks associated with space exploration have a number of relevant counterparts on Earth. The technologies targeted at mitigating these risks are easily-deployable and multi-use, thus they could feasibly be integrated across a wide range of terrestrial care delivery environments.

Many medical devices that are standard of care trace their origins to technologies pioneered or refined by aerospace experts. For example, the very concept of telemetric monitoring was prompted by the desire to send astronaut health information from space to ground control during Project Mercury (72) and has since resulted in ubiquitous terrestrial use, not the least of which is routine intensive care unit (ICU) monitoring. Other recent examples include improved LASIK® eye surgery precision due to innovative optics developed for the Webb telescope (73); materials engineering advances for Martian sample collection that led to biocompatible surgical suture materials (74); and an endoscopic robotic surgery arm inspired by robotic repair arms on the International Space Station (ISS) (75). Clearly, humankind’s extraterrestrial aspirations have stimulated numerous advances across many sectors; here, we focus on medical technologies.

## 4. Space-adapted diagnostic and therapeutic technologies

Early and accurate diagnosis and treatment of health conditions arising during spaceflight can potentially halt or even reverse their negative effects, without requiring a costly, mission-compromising return to Earth or delayed post-flight medical care. As such, easily-deployable and non-invasive diagnostics and therapeutics are prioritized. In this section, we survey recent space-related diagnostic (Supplementary Table S1) and therapeutic (Supplementary Table S2) technologies.

### 4.1. Diagnostics

Imaging and blood analysis comprise two fundamental categories of diagnostic testing. Ultrasound is the primary imaging modality on the ISS due to its versatility, portability, and limited hazards. As a result, its already-numerous applications have been exponentially expanded and include the ability to characterize renal stones (26), vascular flow alterations (27), and bone architecture disorders (28, 29). While the brain remains relatively inaccessible to ultrasound, retinal imaging has been proposed as an alternative to the traditional, and currently space-impractical, CT and MRI modalities for diagnosing stroke (30). The ever-growing library of diagnostic images has concurrently resulted in image-based clinical decision support (CDS) systems too (64). The use of blood component analysis in diagnosis is pervasive, but standard laboratory analyte platforms are unworkable in microgravity, for many reasons including changes in fluid behavior. As a result, techniques that preserve accuracy and facilitate component miniaturization have prevailed, including for CBC (31), WBC differential (32), and multiple electrolyte analysis (33–37).

Unlike portable diagnostics used for intermittent testing, wearable and contactless sensors allow for continuous monitoring. These sensors can be light-, chemical-, current-, or motion-based, implying that the spaces to which they could foreseeably be adapted are numerous. For example, near-infrared spectroscopy (NIRS) relies on analysis of absorption and scatter of specific wavelengths to infer various parameters, such as hemoglobin oxygenation and deoxygenation, blood flow, and presence of water. Transcranial NIRS via headband-like devices can record cerebral hemodynamics during daily activity (15), can identify intracranial edema (16), and can be used in self-deployable polysomnography systems (15, 16). Transdermal NIRS can measure metabolic rate, stroke volume, peripheral hematocrit, and oxygen saturation (38, 39). Other skin-based sensors can detect nutrients, metabolites, and hormones in perspiration (40) or protease activity changes related to muscle atrophy, HIV, and even certain cancers (41). The versatility of these sensors lend them to easy incorporation into mobile monitoring garments, for astronauts and patients alike. Comfortable, washable, wearable, waterproof device-integrated clothing is already available (42–44).

The targets of contactless sensors are largely cognitive and can detect aspects of team cohesion, mental health, sleep deprivation, stress, fatigue, and performance. These devices use a variety of techniques, allowing them to blend into home or work environments, including optical computer facial recognition algorithms (17), radio wave sensors (18), and acoustical analysis (19, 20). Non-invasive biomechanical sensors can also record physical exertion (45), in order to mitigate injury risk during task performance. Overall, environmental enhancements that unobtrusively extract physiologic information from subjects provide accessible, compliance-independent data that can inform decision-making on many scales.

### 4.2. Therapeutics

While the ability to improve early diagnosis of physical and cognitive decrements during long-duration spaceflight is useful, the inability to return to Earth in a reasonable treatment time frame requires the development of portable, easily-deployable therapeutics. In order to minimize payload and maximize multi-use, many of the devices used in diagnosis have also been studied as therapies. Nevertheless, some therapies are consumable and will need to be resynthesized during long-duration missions – a complex problem also addressable with innovative technologies.

Given its portability and extensive diagnostic capabilities, ultrasound technology has also been studied for therapeutic applications, including renal stone propulsion (46), targeted hemostasis (47), and bone remineralization (48). Additionally, wearable ultrasound can treat back pain (49) and potentially provide functional neuromodulation (23) for neurologic and psychiatric disorders. Improvement of sleep and performance have also been trialed using sound stimulation (50–52), light stimulation (53), transdermal vagus nerve stimulation (24), and transcranial NIRS-based photobiomodulation (25).

While these technology-based therapies may supplant standard treatment for some conditions, pharmaceuticals remain critical to the therapeutic arsenal. Radiation sickness and radiation-induced cellular damage is of utmost concern for long-duration extraplanetary missions, resulting in accelerated development of pharmacologic treatment options, including synthetic genistein (65–67) and melanin-based radioprotectants (68–71). However, in addition to human exposure concerns, cosmic radiation also risks degrading pharmaceutical stores (76) and, with the prospect of very limited and very expensive resupply missions, the need to regenerate pharmaceutical compounds is paramount. *In-situ* resource utilization (ISRU) using modified terrestrial biological catalysts along with compounds available on extraplanetary missions to synthesize pharmaceuticals is likely feasible (54–56). Other consumable but life-supporting therapies include intravenous resuscitative fluids, such as crystalloid solutions and blood products. Generation of sterile normal saline from potable water (59) and reconstitution of desiccated, shelf-stable hemoglobin-based oxygen carriers (HBOC) (60) are reasonable solutions for resuscitation in under-resourced conditions.

Finally, integration of CDS with these diagnostic and therapeutic technologies, to streamline and optimize care delivery, is also being tackled by aerospace groups. Integration of structured documentation with mobile critical care modules can ease user burden and improve care (61). Just-in-time training (JITT) with (57) and without telementoring (58) can also improve diagnosis by non-medical personnel. Mixed reality-guided checklist-based software for medical training and real-time management (62, 63) and semi-autonomous and autonomous intelligent agents can facilitate CDS, care coordination, and care delivery (21, 22). Overall, safe advanced care delivery in the remoteness of deep space is approaching a possible reality.

## 5. Elevating terrestrial medical care delivery with aerospace technologies

Given the breadth of biological targets and the versatility of the medical technologies developed for space exploration, finding direct terrestrial applications is not difficult. Integration of these technologies across the patient care spectrum is immediately feasible and is critical to launch medicine into an era of unprecedented access to patient-centered health optimization.

### 5.1. Out-of-hospital applications

**Figure 1:**
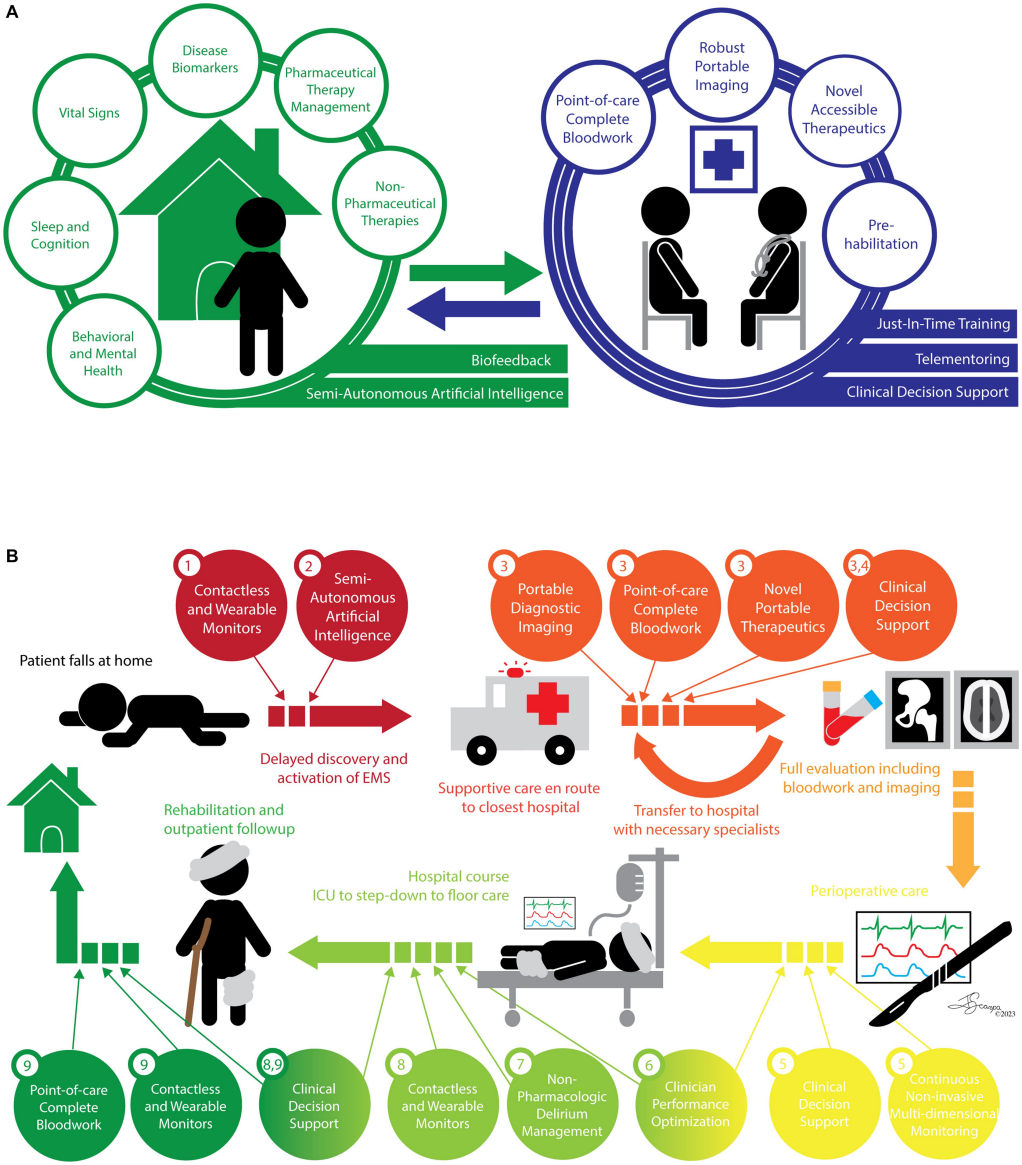
Integration of space-adapted medical technologies in a healthcare workflow model. Two patient care scenarios are presented – an outpatient with chronic illness and an acute surgical emergency. While they ultimately require different management, the integration of space-adapted medical technologies not only improves the efficacy and efficiency of patient care, thereby improving outcomes, quality of life, and patient satisfaction; they can also decrease facility-related length of stay, and thus decrease costs and complication rates. **(A)** Chronic Medical Management. Patient-centered health and wellness can be supported with various contactless and wearable devices that can intelligently monitor a variety of physiologic parameters and provide feedback to the patient and their local provider, empowering them to regulate their health outcomes and intervene early and effectively at home and in their local clinic. Local providers armed with novel technologies to diagnose and treat conditions typically requiring escalation of care can be supported virtually to further facilitate de-centralized, high-quality, and accessible healthcare and wellness optimization. **(B)** Surgical Emergency. An acute event occurs. The standard cycle of care is illustrated and captioned, including a number of gaps and delays that contribute to additional patient morbidity. Widespread integration of space-adapted medical technologies across the care cycle could dramatically improve patient outcomes, quality of life, and costs in a variety of ways including: (1) providing baseline health information and last known well, (2) contributing to timely escalation of care, (3) decreasing time to diagnosis and targeted treatment, (4) improving care coordination by decreasing and streamlining transfers of care, (5) providing multi-dimensional continuous monitoring supported by CDS to rapidly and effectively correct deviations, (6) optimizing clinician performance through non-invasive behavioral and cognitive support technologies, (7) leveraging non-pharmacologic interventions to more effectively prevent and treat in-hospital delirium, (8) facilitating early mobility and safe care de-escalation with unobtrusive patient-centered monitoring including of medical conditions and physical loading, and (9) moving patient care out of facilities and into the home and local clinics so patients can get back to their lives sooner, safely, and in better health.

Management of health, wellness, and chronic illness in the outpatient setting is multi-faceted and is (too) frequently escalated beyond primary care services. Aerospace medical technologies have the potential to keep patients healthier at home and decrease preventable hospitalizations. Integration of these technologies in the home and clinic (Figure 1A) can hasten the decentralization of medical care without sacrificing safety or quality, empowering patients to control their health outcomes and their quality of life.

“Smart” devices and homes are prevalent and ever-growing, but aerospace-derived technologies can fine-tune this growth by expanding biological targets of monitoring, liberating users from traditional workflow constraints, and facilitating care coordination. These intrinsic advantages of aerospace technologies stem from their highly stringent requirements favoring multi-functionality, unobtrusiveness, and integration. Examples of unobtrusive multi-functionality include concurrent, non-invasive monitoring of vital signs, sleep parameters, gait analysis and certain behaviors, like medication compliance and fall detection, to provide valuable feedback for behavioral modification and wellness optimization. Additionally, non-invasive, continuous monitoring of a various patient-specific parameters – such as biomarkers of anxiety and depression, sleep disorders, renal disease, endocrine disorders, and hematologic and oncologic disorders – would limit the need for repeated visits to clinics for invasive, expensive bloodwork. This can also provide unbiased, compliance-independent, and high-density information about disease states, including the efficacy of prescribed interventions and relevant outcomes of clinical trials. Portable and resilient point-of-care bloodwork devices can facilitate testing in clinics or at home, supporting medication titration and side-effect management without needing to travel to the office. Finally, in addition to providing readily available, structured medical and mental health support at home, semi-autonomous conversational intelligent agents can also escalate and coordinate care resources as needed, resulting in timely intervention.

Aerospace technologies are also primed for expanding clinical access by empowering non-expert providers to safely, confidently care for numerous conditions with assistance from AI-CDS, JITT, and specialist telementoring, in a model similar to non-physician astronauts caring for their fellow spacefarers. Expansion of remotely-guided and AI-assisted ultrasound-based diagnostics and therapeutics is a game-changing possibility for care delivery in under-resourced areas (77). These technologies can decrease hospitalizations by allowing earlier detection of disease and earlier intervention, even (and especially) by non-specialists. Treatment of certain ailments that previously required care escalation could initially be attempted in the home or office, such as ultrasound-based renal stone management or hemorrhage control. Similarly, infusion of shelf-stable reconstituted HBOCs could obviate the need to travel long distances for patients requiring transfusion. In cases of extreme remoteness or other travel impediment, such as in pandemics or wars, ISRU and synthetic pharmacy could restore stocks of basic medical supplies.

Furthermore, these technologies not only have the potential to improve baseline health, they can also improve specific chronic conditions ahead of elective surgery, decreasing perioperative morbidity. At-home data collection could also inform preoperative and risk evaluation, providing patients and clinicians with individualized risk–benefit assessments. “Pre-habilitation” programs for at-risk patients could include biometric and metabolic/hormone monitoring to support targeted systemic disease optimization and NIRS-based photobiomodulation to shore up cognitive and psychological reserve ahead of surgery.

Last, but not least, device detection of deviations from known baseline and semi-autonomous contacting of a clinician or emergency services can limit delays in care, especially for time-sensitive conditions like stroke or myocardial infarction. Emergency services equipped with portable or wearable blood component or perspiration analysis devices, advanced diagnostic and therapeutic ultrasound, retinal imaging, and AI-CDS could more rapidly diagnose specific critical conditions, initiate earlier targeted treatment *en route* to a hospital, and redirect transport to a specialty hospital based on tentative technology-assisted diagnoses, limiting the need for late transfers of care. Overall, aerospace medical technology can rapidly bring 21st century medicine to all patients.

### 5.2. In-hospital applications

Translation of space-adapted medical technologies is primed for high-acuity hospital areas (Figure 1B). Faster, more accurate diagnostics in the Emergency Department (ED), especially for critically ill or unstable patients would undoubtedly aid decision-making and treatment. Integration of the above portable blood testing platforms and ultrasound-based diagnostics to the standard arsenal should be prioritized. Furthermore, use of ultrasound-based therapeutics in this setting, such as for hemostasis or kidney stone propulsion, should be considered.

ICU care could be augmented by space-adapted technologies. For example, patients could be non-invasively and continuously monitored for muscle degradation (from being bed-bound) or improvement (from physical therapy), drug metabolites (especially of sedatives or narcotics), and cancer progression (given treatment is paused for ICU admission), without multiple painful, costly blood draws. Stroke could be identified using retinal imaging without needing urgent and unwieldy transport to the CT scanner. Nutritional supplementation decisions could be supported with NIRS-based measurements of metabolic rate. Stroke volume and hematocrit could also be monitored by NIRS. Circadian rhythm disorders could be averted or treated with non-pharmaceutical sleep entrainment, reducing delirium and sedative use. Seamless multi-modal physiologic monitoring, including brain monitoring, could assist with mental health and delirium assessment, sleep monitoring, drug dosing, or detection of disease state or acute changes; their portable nature can mitigate the cumbersomeness of patient transport to procedures and imaging, and can improve periprocedural care by limiting the need to connect and disconnect monitoring multiple times.

Unobtrusive physiologic monitoring can also facilitate patient care transfers and patient mobility within the hospital. Sensor-equipped patient gowns or wearables could allow continuous, yet comfortable, monitoring; coupling these continuous inputs with CDS could track patient clinical status longitudinally and determine transfers to different levels of care. CDS can also integrate the multi-modal data to better direct which procedures and interventions are offered and reduce unrecognized bias in care delivery (78). Wearables deployed during acute changes in status would facilitate monitoring of the patient during resuscitation, triage, and transport. Finally, sensor-equipped gowns could improve patient mobility during hospitalization while maintaining a safety net of monitoring, improving physical and mental health rehabilitation and patient satisfaction.

Ultimately, the timely and safe de-escalation of care acuity can be facilitated with these technologies, including transitions to non-hospital facilities. For example, non-invasive sensors of metabolic rate, skeletal muscle mass, and cardiac parameters could be of great utility in monitoring physical therapy progress as well as physiologic parameters that would limit the extent of therapy in at-risk patients. Wearable unobtrusive monitors could provide a virtual safety net supported by telementoring and consultancy medicine, keeping patients healthier and out of the hospitals as much as possible.

### 5.3. Clinician performance and wellness applications

Finally, space-adapted medical technologies have the potential to improve clinician performance and wellness. Application of biomedical sensors to the patient care environment have the potential to record physical loading and torque experienced by staff such as nurses, physical and occupational therapists, or patient care assistants during routine physical tasks required for patient care; this can help inform and improve workplace safety measures to prevent injury as well as improve patient safety. Other “smart” workplace design possibilities would include incorporation of contactless monitoring – optical, acoustical, radio wave detection – of cognitive or mental health decrements in high-stakes, stressful environments, such as the ED, OR, or ICU. Real-time unbiased feedback on patient safety and team dynamics can speed the quality improvement cycle.

Much like astronauts, clinicians can experience cognitive decrements resulting from heightened stress and decreases in sleep quantity or quality. In particular, clinicians working in complex environments like the ED, the OR, or the ICU, where heightened vigilance is required and emergency action is not uncommon, are at risk for suboptimal attention and performance despite maximal effort, especially during overnight or 24-h shifts. The same could be said for care delivery personnel operating in remote or under-resourced locations. Enhancing cognition and performance during these shifts, and enhancing *recovery* after these shifts, may improve clinician well-being, reduce burnout, and improve outcomes. Routine availability or application of non-pharmaceutical sleep-improving or performance-boosting technologies for medical practitioners either in the workplace (e.g., while on call) or at home is worth exploring.

## 6. Conclusion

Disruptive medical technologies, like those required to support extraplanetary human existence, have an extensive array of potential applications across all terrestrial care delivery settings, for both patients and practitioners. Rapid and efficient clinical validation of these technologies and their integration into standard clinical workflow must be a priority and requires the involvement of clinicians to spearhead the charge. The future of democratized, safe, quality healthcare delivery is already here.

## Data availability statement

The original contributions presented in the study are included in the article/Supplementary material, further inquiries can be directed to the corresponding author.

## Author contributions

JS wrote the first draft of the manuscript. All authors contributed to the scope and content of the manuscript, read, edited, and approved the final submitted version of the manuscript.

## Conflict of interest

SP is employed by company Fluidity Technologies, Inc. SP is employed by Operator Solutions and Voyager Space Holdings as a board member.

The remaining authors declare that the research was conducted in the absence of any commercial or financial relationships that could be construed as a potential conflict of interest.

## Publisher’s note

All claims expressed in this article are solely those of the authors and do not necessarily represent those of their affiliated organizations, or those of the publisher, the editors and the reviewers. Any product that may be evaluated in this article, or claim that may be made by its manufacturer, is not guaranteed or endorsed by the publisher.

## Supplementary material

The Supplementary material for this article can be found online at: https://www.frontiersin.org/articles/10.3389/fmed.2023.1226531/full#supplementary-material

## Glossary

AI: Artificial Intelligence
AMRA: Autonomous Medical Response Agent
CBC: Complete Blood Count
CDS: Clinical Decision Support
CMO: Crew Medical Officer
CT: Computed Tomography
ECG: Electrocardiography
ED: Emergency Department
EEG: Electroencephalography
HBOC: Hemoglobin-Based Oxygen Carriers
ICU: Intensive Care Unit
ISS: International Space Station
ISRU: *In-Situ* Resource Utilization
IV: Intravenous
JITT: Just-In-Time Training
MRI: Magnetic Resonance Imaging
NASA: National Aeronautics and Space Administration
NIRS: Near-Infrared Spectroscopy
PACU: Post-Anesthesia Care Unit
SANS: Spaceflight-Associated Neuro-ocular Syndrome
WBC: White Blood Cell
