# Supplementary material for: Space exploration as a catalyst for medical innovations
Source: Front Med (Lausanne). 2023 Jul 19;10:1226531. doi: 10.3389/fmed.2023.1226531 (PMC10395101; doi:10.3389/fmed.2023.1226531)
Supplement: Supplementary file 1 [file Data_Sheet_1.docx]

**Supplementary Figure 1. Microgravity Effects on Human Physiology.** Prolonged exposure to microgravity affects all organ systems, with variable trajectories, plateaus, and reversibility upon return to Earth. The combined decline of these organ systems may ultimately result in physiology more comparable to that of a critically-ill patient, making medical planning and care delivery, even for relatively minor conditions, that much more complex in the remoteness of deep space.

*
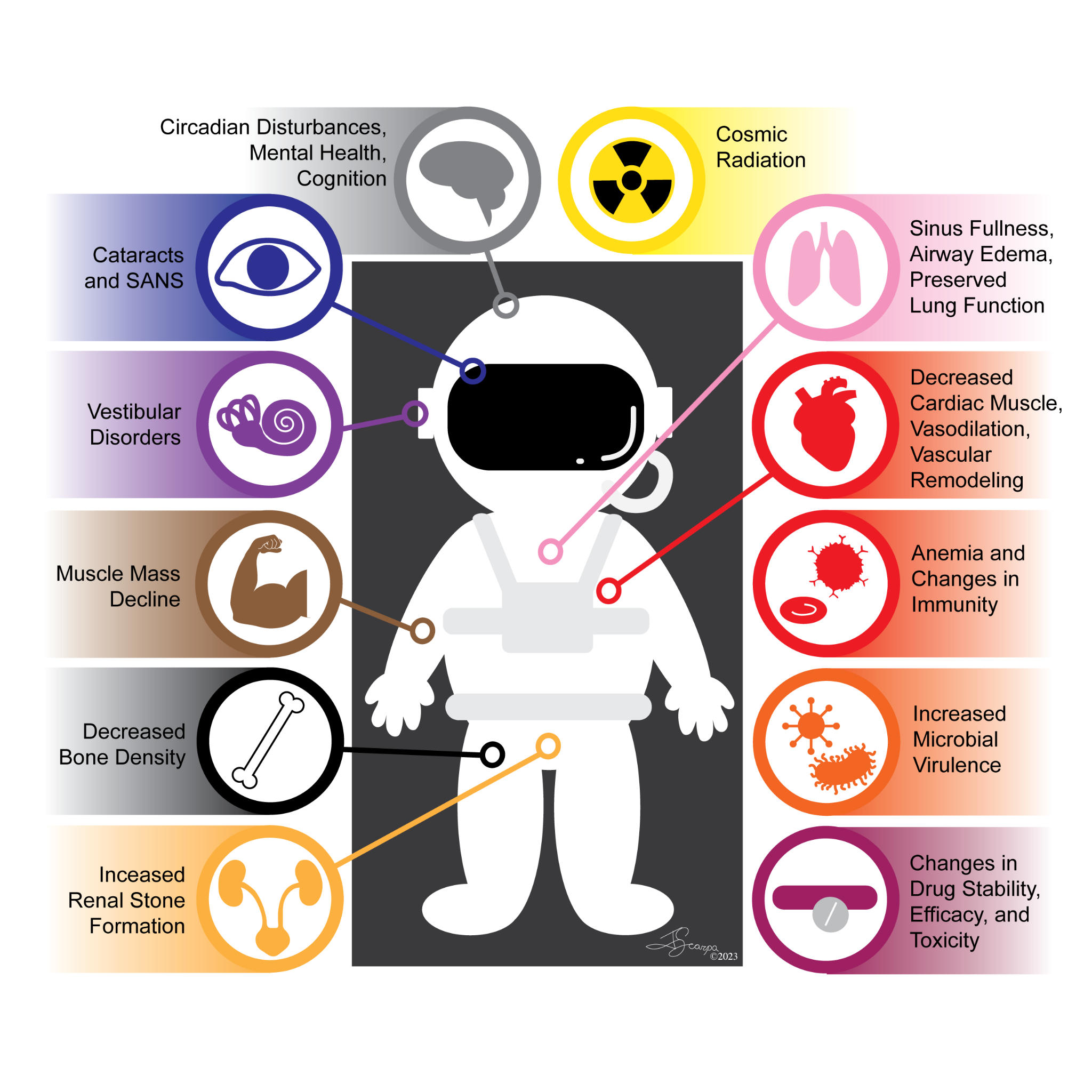
*

**Supplementary Table 1. Portable, Wearable, and Contactless Diagnostics.**

|  | **Technology** | **Description** | **Potential Applications** | **References** |
| --- | --- | --- | --- | --- |
| **Portable** | Ultrasound imaging | Diagnosis and characterization of renal stones using Doppler twinkling artifact and B-mode ultrasound | - Early diagnosis of renal stones can decrease time to treatment by limiting the need for hospital referral or advanced imaging (X-ray, CT) - Characterization of renal stone type can inform clinical decision making and patient counseling | Sorensen *et al.* (2013)^19^ |
|  |  | Identification of vascular flow and structural alterations in the setting of total body fluid shifts | - Characterization of blood flow anomalies in disease states with altered fluid handling | Marshall-Goebel *et al.* (2019)^20^ |
|  |  | Characterization of trabecular bone structure using pulse- and frequency-modulated ultrasound | - Diagnosis of bone disorders, including osteopenia and osteoporosis - Determination of treatment efficacy and disease progression for bone disorders | Lin *et al.* (2009)^21^  TRS Ceramics, Inc.^22^ |
|  | Retinal imaging | Rapid alternative modality for the diagnosis of ischemic versus hemorrhagic stroke, as an alternative to bulky CT/MRI | - Rapidly diagnose and initiate stroke treatment in the prehospital setting - Diagnose and initiate stroke treatment in the ICU without needing transport to advanced imaging (CT, MRI) | Pachade *et al.* (2022)^23^ |
|  | Blood component analysis | Ultra-compact, rapid CBC analysis from microliter blood samples | - Rapid diagnosis of bleeding, infection, and platelet disorders in emergency, urgent, remote, or disaster settings - Clinic- or home-based testing to monitor disease progression or treatment effects, including for cancer, blood disorders, or infections | CytoTracker by RizLab Health^25^ |
|  |  | Portable determination of five-part WBC differential and analysis of multiple WBC subtypes | - Point-of-care monitoring for sepsis, HIV, and certain cancers | LeukoDx, Inc.^26^ |
|  |  | Multiple electrolyte analysis using miniaturized physical components or repurposing of existing technology | - Rapid diagnosis in emergency, urgent, remote, rural, extreme, under-resourced, disaster, or pandemic settings - Clinic- or home-based testing to monitor disease progression, treatment effects and drug side effects across many disease states - Monitoring to inform home- or clinic-based drug titration | Intelligent Optical Systems, Inc.^27^  The DNA Medicine Institute^28^  Ionu Biosystems^29^  Ativa Medical^30^  InnaMed^31^ |
| **Wearable** | Near-infrared spectroscopy (NIRS) | Long-term transcranial NIRS monitoring with simultaneous multi-modal physiologic parameters | - Detection of circadian rhythm transitions and cognitive performance alterations resulting from sleep restriction or deprivation, such as during prolonged or night shifts - Home-based polysomnography | Zhang *et al.* (2014)^32^ |
|  |  | Transcranial NIRS monitoring of cerebrovascular parameters and intracranial edema | - Emergency, ICU, and perioperative management of intracranial pathologies - Prehospital and remote diagnosis assistance for intracranial pathologies and emergencies | Strangman *et al.* (2018)^33^ |
|  |  | Transdermal NIRS for monitoring of real-time metabolic rate, stroke volume, hematocrit, and oxygen saturation | - ICU management of nutrition, ventilator weaning, cardiac and respiratory diseases, and sepsis - Monitoring and rapid diagnosis of shock types in prehospital, emergency, and disaster settings | Soller *et al.* (2012)^34^  Reflectance Medical, Inc.^35^ |
|  | Electro-chemical biosensor | Real-time detection of nutrients, metabolites, electrolytes, and hormones in perspiration using a flexible array | - Home- or clinic-based monitoring of stress, anxiety, drug compliance or side effects, disease progression or treatment effects - Perioperative and ICU management of nutritional, metabolic, hormonal, and electrolyte derangements without the need for multiple invasive testing | Wang *et al.* (2022)^36^ |
|  | Voltammetric analysis | Nanoelectrode array chip-based voltammetry to detect protease activity changes | - Monitoring muscular atrophy, HIV, and even certain cancers, as well as treatment effects - Monitoring of hospital-related muscle disuse and physical therapy countermeasures | Anderson *et al.* (2020)^37^ |
|  | Clothing-based sensors | Sensors incorporated into comfortable bionic garments | - Liberating patients from wires to allow earlier and easier mobility, increased satisfaction, and easier patient transport - Remote monitoring in the home or non-hospital facilities | Nyx Illuminated Clothing^38^  NanoSonic, Inc.^39^  Orbital Research, Inc.^40^ |
| **Contact less** | Clinical decision support (CDS) | CDS for ultrasound-based diagnoses by non-experts | - Diagnostic assistance in rural, remote, or under-resourced areas, or during scenarios requiring non-expert care, like pandemics or disasters | VisualDx^24^ |
|  | Facial recognition | Detection of emotions, stress, fatigue, and performance with pose-robust optical facial detection and tracking | - Diagnostic assistance for detection of pain and psychological distress - Monitoring and detection of performance, stress, and fatigue in high-stress, high-vigilance environments (ED, OR, ICU, PACU, disaster or pandemic settings) | Yu *et al.* (2016)^41^ |
|  | Radio waves | Extraction of behavior, mobility, and sleep parameters from analysis of reflected radio wave signals | - Monitoring performance, medication compliance, patient safety, and sleep behavior - Home-based mobility and sleep monitoring and feedback | Emerald Innovation^42^ |
|  | Acoustical analysis | Detection of motor and cognitive decrements, fatigue, stress, anxiety, and depression from content-independent acoustical analysis of speech | - Early diagnosis and monitoring of disease progression for neurological disorders including Parkinson’s and Alzheimer’s diseases - Monitoring and detection of decrements in high-stress high-vigilance environments (ED, OR, ICU, PACU, disaster or pandemic settings) | Lieberman *et al.* (2005)^43^  Driskell *et al.* (2021)^44^ |
|  | Bio-mechanical sensors | Monitoring of physical exertion and load dynamics | - Mitigation of injury risk during physical task performance, e.g. physical therapists, beside nurses, patient care assistants | Vitali *et al.* (2020)^45^ |

**Supplementary Table 2. Portable, Wearable, Contactless, and Regenerable Therapeutics.**

|  | **Technology** | **Description** | **Potential Applications** | **References** |
| --- | --- | --- | --- | --- |
| **Portable** | Radio-protectants | Oral and parenteral administration of synthetic and naturally-derived isoflavone- and pigment-based compounds decrease acute radiation effects | - Pre-treatment for cancer radiotherapy to mitigate side effects and allow for increased targeted radiation exposure - Post-exposure treatment for nuclear disasters or other unintentional radiation exposure | Singh *et al*. (2023)^57^  Li et al. (2022)^58^  Salem *et al.* (2022)^59^  Revskaya *et al.* (2012)^60^  Malo *et al.* (2022)^61^  Kunwar *et al.* (2012)^62^  Schweitzer *et al.* (2010)^63^ |
|  | Ultrasound | Short bursts of ultrasonic pulses safely reposition renal stones transcutaneously | - Office-based non-invasive treatment of renal colic - Pre- or intra-procedural positioning assistance for standard ureteroscopy or shock wave lithotripsy | May *et al.* (2016)^46^ |
|  |  | High-intensity focused ultrasound achieves (arterial) hemorrhage control with long-term hemostasis and preservation of flow and vessel structure | - Prehospital trauma management - Perioperative applications in trauma and other high-risk patients - Temporizing and definitive care in remote, under-resourced, or disaster-stricken areas | Zderic *et al.* (2006)^47^ |
|  |  | Low-intensity amplitude-modulated pulsed ultrasound increases osteoblastic mineralization | - Home- or clinic-based non-pharmacologic osteopenia and osteoporosis treatment - Accelerating bone healing post-orthopedic surgery | Uddin *et al.* (2010)^48^ |
| **Wearable** | Ultrasound | Long-duration, low-intensity, wearable ultrasound improves back pain related to herniated nucleus pulposus | - Non-pharmacological primary or adjuvant treatment for back pain, especially for patients with limited healthcare access - Accelerating healing of certain musculoskeletal injuries | ZetrOZ Systems^49^ |
|  |  | Low-intensity transcranial focused ultrasound produces regional functional neuromodulation | - Non-pharmacological adjuvant treatment for neurological and psychiatric disorders, to mitigate side effects from pharmaceuticals | Kim *et al.* (2022)^50^ |
|  | Acoustic stimulation | Sound stimulation within the audible spectrum during sleep increases slow waves | - Improving short-duration sleep and performance after awakening while on-call or during overnight shifts - Hospital- and ICU-associated delirium prophylaxis and treatment | Tononi *et al.* (2010)^51^  SmartSleep^52,53^ |
|  | Light stimulation | Millisecond pulsed visible light through an eye mask speeds circadian realignment without disrupting sleep | - Improving restorative sleep following prolonged or overnight shifts and increasing alertness after wake up - Hospital- and ICU-associated delirium prophylaxis and treatment | LumosTech, Inc.^54^ |
|  | Transdermal vagus stimulation | Cervical transcutaneous vagal nerve stimulation following prolonged wakefulness decreases fatigue and increases arousal and multitasking performance | - Improving alertness and performance during prolonged or overnight shifts - Improving alertness and performance during complex or high-risk procedures | McIntire *et al.* (2021)^55^ |
|  | Photo-biomodulation | Transcranial near-infrared stimulation increases functional connectivity of illuminated regions and across the brain | - Boosting cognitive performance - Treating cognitive disorders, including dementias | Dmochowski *et al.* (2020)^56^ |
| **Contactless** | Integration of medical documentation and medical support | Fully integrated mobile critical care patient support and medical documentation, including semi-autonomous capabilities and AI-CDS | - Prehospital care - Natural disasters and humanitarian crises - Pandemics and other expansion of critical care needs | iRevive-Lightweight Trauma Module^70^ |
|  | JITT and telementoring | Preflight basic training couple with onboard virtual refreshers and telementoring allows non-medical crew to perform medical tasks successfully | - Expanding the availability of diagnostics and procedures to remote, rural, or under-resourced areas by supporting local providers - Supporting diagnostics and procedures performed in the field during emergencies | Foale *et al.* (2005)^71^  Hurst *et al.* (2015)^72^  Kirkpatrick *et al.* (2013)^77^ |
|  | Assistive and semi-autonomous artificial intelligence | Augmented reality-based assistant can provide procedural guidance and CDS | - Procedural skills learning for trainees or for non-experts, such as in remote or under-resourced environments - Procedural planning and virtual practice for experts performing rare or highly complicated procedures | Burian *et al.* (2023)^73^  Ebnali *et al.* (2022)^74^ |
|  |  | Conversational intelligent agent with autonomous capabilities can promote physical and behavioral health | - Home-based health monitoring and behavioral modification, such as improving medication compliance and providing at-home talk therapy | TRISHA by Ejenta^75^ |
|  |  | Autonomous medical response agent can assist with diagnosis and treatment, and can coordinate care with remote consultants | - Home-based care assistance - CDS and consultancy support for primary care providers or non-specialists in remote areas | AMRA by Nahlia, Inc.^76^ |
| **Regenerable** | Sterile crystalloid generation | IVGen can purify existing potable water sources and add electrolytes to create sterile intravenous normal saline | - Fluid resuscitation in under-resourced and remote settings - Supply supplementation during shortages, disasters, or pandemics | McQuillen *et al.* (2011)^68^ |
|  | Synthetic blood products (HBOCs) | Synthetic, desiccated red blood cell substitutes can be reconstituted on demand and administered for hemorrhage resuscitation, providing oxygen delivery to tissues | - Massive hemorrhage - Out-of-hospital hemorrhage - Emergency use when cross-matched blood is not yet available - Transfusion for patients with rare blood types or with multiple antibodies - Chronic transfusions, in order to avoid generation of multiple antibodies - Transfusion in remote, extreme, or under-resourced areas | ErythroMer by KaloCyte^69^ |
|  | ISRU and pharmaceutical synthesis | Synthesis of pharmaceutical compounds using in-situ resources and biotic or abiotic methods can replenish expired, irradiated, or depleted drug stocks | - Supply supplementation during shortages, disasters, or pandemics - Basic drug stocking in remote, extreme, or under-resourced areas | Menezes *et al.* (2015a)^65^  Menezes *et al.* (2015b)^66^  McNulty *et al.* (2021)^67^ |
